# Supplementary material for: The Uremic Toxin Indoxyl Sulfate Accelerates Senescence in Kidney Proximal Tubule Cells
Source: Toxins (Basel). 2023 Mar 25;15(4):242. doi: 10.3390/toxins15040242 (PMC10143766; doi:10.3390/toxins15040242)

Table S1. top 5 significant p-values and adj.p-values for KEGG 2021 Human in each cluster.

Cluster	Term	p-value	adj.p-value	Overlap_genes
A	Cell cycle	1.30E-12	3.02E-10	[CDKN2C, PLK1, BUB1B, TTK, CDC6, CDC25C, PKMYT1, CDC25A, CDC20, CCNA2, CCNB2, CCNB1, ORC6, DBF4, PTTG1, ORC1, ESPL1, CDK1, E2F2, SFN, BUB1, MAD2L1]
	Oocyte meiosis	1.56E-06	1.81E-04	[PLK1, CDC25C, FBXO43, PKMYT1, AURKA, CDC20, SGO1, CCNB2, CCNB1, PTTG1, ESPL1, CDK1, FBXO5, BUB1, MAD2L1]
	Progesterone-mediated oocyte maturation	1.24E-05	9.64E-04	[CCNA2, CCNB2, CCNB1, PLK1, CDK1, KIF22, CDC25C, PKMYT1, BUB1, CDC25A, AURKA, MAD2L1]
	Gap junction	1.93E-05	1.12E-03	[TUBA1C, TUBA1B, TUBB6, TUBB2B, GUCY1B1, TUBA1A, TUBB2A, TUBB, CDK1, TUBB4B, TUBB4A]
	Salmonella infection	9.63E-04	4.49E-02	[ANXA2, TUBB, TUBB4B, TUBB4A, TNF, PIK3CG, TUBA1C, TUBA1B, TUBB2B, TUBB6, TUBA1A, TUBB2A, IRAK1, PODXL, RHOJ, S100A10]
C	p53 signaling pathway	3.80E-05	9.71E-03	[PIDD1, CDKN1A, RRM2B, ZMAT3, SESN1, SESN2, ADGRB1, MDM2, BBC3, DDB2]
	NF-kappa B signaling pathway	1.75E-04	2.23E-02	[PIDD1, VCAM1, TNFSF14, BCL2A1, TNFAIP3, CXCL3, PTGS2, CXCL2, CARD11, EDA2R, BIRC3]
E	Protein digestion and absorption	2.00E-06	6.45E-04	[COL15A1, COL16A1, MME, COL25A1, COL11A1, COL12A1, SLC1A1, SLC16A10, ATP1B2, ATP1B1, COL1A2, COL6A2, COL7A1, COL4A6, COL8A2, COL4A5, COL8A1, COL6A5]
	Cell adhesion molecules	3.34E-04	3.91E-02	[CD274, NTNG2, CADM1, NEGR1, SDC2, HLA-B, HLA-A, F11R, HLA-F, L1CAM, HLA-G, CLDN5, PECAM1, ITGB8, NRCAM, CLDN16, TIGIT, JAM2]
	Human papillomavirus infection	4.26E-04	3.91E-02	[NOTCH3, TNXB, WNT2B, NOTCH4, FOXO1, OASL, PARD6B, CCND2, WNT11, ITGB8, WNT2, FZD3, WNT5B, STAT1, MX2, WNT5A, ITGA1, HLA-B, HLA-A, AXIN2, HLA-F, HLA-G, VEGFA, COL1A2, DLG3, COL6A2, COL4A6, COL4A5, ITGA7, MAML3, COL6A5]
F	Cytokine-cytokine receptor interaction	1.31E-08	3.00E-06	[IL33, CXCL6, CSF3, CCL20, GDF15, EBI3, IL24, CXCR4, TNFRSF11B, TNFRSF1B, CX3CL1, IL6, IL36B, IL1B, CCL4, CCL3, ACKR3, IL36RN]
	Complement and coagulation cascades	5.76E-08	6.00E-06	[C3, THBD, C1R, C5AR1, BDKRB2, ITGAX, BDKRB1, C8B, C8A, CLU]
	Viral protein interaction with cytokine and cytokine receptor	2.74E-07	1.80E-05	[CXCL6, IL6, CCL20, IL24, CCL4, CCL3, CXCR4, ACKR3, TNFRSF1B, CX3CL1]
	Inflammatory bowel disease	1.07E-05	5.34E-04	[IL6, IL1B, RORC, STAT4, RORA, NOD2, TLR2]
	Amoebiasis	2.65E-05	9.10E-04	[IL6, GNA15, MUC2, IL1B, CD1D, C8B, C8A, TLR2]

Table S2. Top 5 significant p-values and adj.p-values for MSigDB Hallmark 2020 in each cluster.

Cluster	Term	p-value	adj.p-value	Overlap_genes
A	G2-M Checkpoint	5.93E-57	2.79E-55	[TOP2A, H2AX, JPT1, SUV39H1, CCNF, KIF11, MKI67, BRCA2, SMC4, KIF15, LMNB1, CKS1B, CDC20, PTTG1, EXO1, STMN1, PBK, NUSAP1, NEK2, MYBL2, FBXO5, KPNA2, HMGN2, KIF23, KNL1, KIF22, CDC25A, TRAIP, CCNA2, DDX39A, DBF4, ESPL1, INCENP, CKS2, BIRC5, KIF2C, KIF20B, TROAP, TTK, HMMR, CENPA, AURKB, AURKA, CCNB2, ORC6, RACGAP1, E2F2, BUB1, PLK4, H2AZ1, GINS2, POLQ, STIL, CDKN2C, UBE2C, PLK1, CDC6, NDC80, TPX2, CENPE, CENPF, UBE2S, PRC1, KIF4A, TACC3, CDK1, MAD2L1, CDKN3]
	E2F Targets	5.53E-53	1.30E-51	[TOP2A, H2AX, JPT1, SUV39H1, DSCC1, HMGB2, BUB1B, MKI67, BRCA2, SMC4, CKS1B, LMNB1, CDC20,PTTG1, STMN1, MYBL2, TK1, KPNA2, DLGAP5, RFC3, TUBB, KIF22, CDC25A, NME1, DDX39A, MELK, ESPL1, CKS2, DEPDC1, TIMELESS, BIRC5, KIF2C, DCTPP1, ASF1B, CDCA3, CDCA8, HMMR, AURKB, AURKA, RAD51AP1,CCNB2, ORC6, RACGAP1, LYAR, PLK4, GINS1, H2AZ1, CDKN2C, RRM2, SPAG5, GINS3, PLK1, GINS4, CENPE, KIF18B, DIAPH3, UBE2S, KIF4A, TACC3, CDK1, TRIP13, SPC24, MAD2L1, CDKN3, SPC25]
	Mitotic Spindle	3.51E-19	5.50E-18	[TOP2A, PIF1, TTK, KIF11, BRCA2, SMC4, KIF15, LMNB1, AURKA, CCNB2, RACGAP1, NUSAP1, NEK2, FBXO5,SYNPO, BUB1, DLGAP5, PLK1, KIF23, KIF22, NDC80, ANLN, TPX2, CENPE, CENPF, ESPL1, INCENP, PRC1, KIF4A,CDK1, BIRC5, KIF2C, KIF20B, EZR, DOCK2]
	Spermatogenesis	2.38E-04	2.60E-03	[CCNB2, ARL4A, DBF4, CAMK4, CDK1, NEK2, KIF2C, TTK, BUB1, NCAPH, AURKA, CDKN3]
	Estrogen Response Late	2.77E-04	2.60E-03	[PLK4, TOP2A, GINS2, SLC26A2, STIL, CAV1, CDC6, CDC20, FABP5, ACOX2, PRKAR2B, GJB3, ID2, SFN, KIF20A]
C	TNF-alpha Signaling via NF-kB	5.21E-10	1.04E-08	[BTG2, CDKN1A, CEBPB, CSF2, BCL2A1, CEBPD, TNFAIP3, PTGS2, CXCL3, CXCL2, IFIH1, MAP3K8, ICOSLG,IL15RA, GCH1, GFPT2, SOD2, PNRC1, IL1A, NINJ1, FOSB, PTX3, CD69, PLPP3, BIRC3]
	p53 Pathway	5.21E-10	1.04E-08	[CYFIP2, CDKN1A, BTG2, GLS2, DGKA, HSPA4L, NDRG1, PIDD1, SOCS1, ZMAT3, SESN1, FDXR, CLCA2, CASP1,PHLDA3, ST14, RHBDF2, IRAG2, DDB2, IL1A, KRT17, NINJ1, MDM2, NUPR1, ADA]
	Interferon Gamma Response	1.10E-06	1.47E-05	[IL15RA, CDKN1A, CIITA, VCAM1, GCH1, MX1, TNFAIP3, ISG15, PTGS2, DDX60, SOD2, USP18, IFIH1, SOCS1,IFI27, TNFSF10, CASP1, IRF7, CD69, HERC6]
	Interferon Alpha Response	1.71E-03	1.71E-02	[IFIH1, IFI27, MX1, CASP1, IRF7, ISG15, DDX60, USP18, HERC6]
	Inflammatory Response	4.69E-03	3.75E-02	[NPFFR2, IL15RA, CDKN1A, BTG2, GCH1, LPAR1, RASGRP1, IL1A, RNF144B, TNFSF10, IRF7, CD69, ICOSLG]
D	KRAS Signaling Dn	7.42E-04	2.97E-02	[PRKN, EDN1, TGFB2, EDN2, KRT4, RSAD2, ARPP21, SLC30A3, IGFBP2, ALOX12B, YBX2, MACROH2A2, P2RX6,P2RY4, NTF3, EFHD1, CDH16, GPR19, RIBC2, TAS2R4]
E	Epithelial Mesenchymal Transition	6.92E-16	3.18E-14	[COL16A1, CXCL8, LRP1, COL11A1, COL12A1, PLOD2, FBLN1, CXCL1, PLOD1, ENO2, FSTL1, SAT1, FBLN5, GJA1,QSOX1, SLIT2, TGM2, CTHRC1, CADM1, PRRX1, IGFBP4, MMP1, LUM, FUCA1, MMP2, WNT5A, APLP1, MMP3,BGN, INHBA, VEGFA, GREM1, COL1A2, FAP, LOX, COL6A2, COL7A1, COL8A2, FMOD, MXRA5, SNTB1, FBN1]
	Hypoxia	1.90E-10	4.37E-09	[SCARB1, PFKFB3, SDC2, ADM, VLDLR, ENO2, HK2, CSRP2, FAM162A, STC2, MXI1, PDK3, GPC4, B4GALNT2,PPARGC1A, ANKZF1, PDK1, TGM2, WSB1, DTNA, GAA, BGN, FOS, AMPD3, HS3ST1, VEGFA, EFNA3, PPP1R3C,LOX, P4HA1, DDIT4, RRAGD, ALDOC, TMEM45A]
	Interferon Gamma Response	4.34E-08	6.65E-07	[LGALS3BP, CD274, CFH, C1S, FPR1, SECTM1, IFI44L, OASL, CCL7, NAMPT, DHX58, EPSTI1, ST8SIA4, PDE4B,CCL2, AUTS2, STAT1, MX2, HLA-B, IFI44, ARID5B, HLA-A, HLA-G, BST2, PLSCR1, OAS2, SSPN, CMPK2, XAF1, CFB]
	Estrogen Response Early	1.70E-06	1.96E-05	[SCARB1, FOXC1, FCMR, WWC1, HSPB8, SLC1A1, ABAT, ADD3, SLC7A2, GJA1, PDLIM3, ABLIM1, DEPTOR,RPS6KA2, STC2, TGM2, BCL11B, IGFBP4, GFRA1, FOS, GAB2, IL17RB, DHRS3, AR, ELF3, SYT12, RAB17]
	KRAS Signaling Up	5.33E-06	4.91E-05	[ADAMDEC1, ABCB1, CFH, SATB1, HOXD11, TFPI, HSD11B1, CCND2, ADGRA2, PRKG2, ST6GAL1, JUP, PRRX1,FUCA1, IGF2, LAPTM5, INHBA, TRAF1, RBP4, GPRC5B, MAFB, HKDC1, SCG5, PECAM1, TRIB1, CFB]
F	Inflammatory Response	1.45E-08	5.65E-07	[CXCL6, CSF3, TNFAIP6, CCL20, AQP9, EBI3, C5AR1, NOD2, TNFRSF1B, CX3CL1, IL6, GNA15, IL1B, BDKRB1, TLR2]
	Coagulation	6.94E-07	1.35E-05	[C3, DPP4, THBD, MMP7, GDA, C1R, TFPI2, HTRA1, C8B, CLU, C8A]
	TNF-alpha Signaling via NF-kB	4.38E-06	5.69E-05	[DUSP4, NR4A2, CXCL6, IL6, TNFAIP6, CCL20, IL1B, KYNU, CCL4, SLC16A6, ACKR3, TLR2]
	Epithelial Mesenchymal Transition	2.48E-05	2.42E-04	[LRRC15, CXCL6, IL6, IGFBP3, TFPI2, HTRA1, TNFRSF11B, THBS2, SCG2, NID2, DCN]
	KRAS Signaling Up	1.28E-04	1.00E-03	[IL33, SNAP25, SLPI, FGF9, CCL20, IL1B, IGFBP3, CXCR4, PRDM1, TNFRSF1B]

Table S3. top 10 enrichment pathways from MSigDB Hallmark of DEGs in IS-treatment groups compared to non-treatment groups at different time points.

Group	Term	P-value	Adjusted P-value	Genes
Day1+IS vs Day1	TNF-alpha Signaling via NF-kB	3.69E-22	8.50E-21	CXCL6; CSF2;BCL2A1;GCH1;CCL20;TNFAIP3;CXCL1;CXCL3;PTGS2;CXCL2;IL1A;IL6;NR4A3;CCL2;MSC;ICOSLG;BIRC3
	KRAS Signaling Up	1.47E-09	1.13E-08	GPRC5B;CSF2;ABCB1;CCL20;TNFAIP3;SOX9;PTGS2;EREG;BIR
	Inflammatory Response	1.47E-09	1.13E-08	CXCL6; IL1A;IL6;CXCL8;GCH1;CCL20;CCL2;ICOSLG;EREG
	Epithelial Mesenchymal Transition	3.26E-08	1.87E-07	CXCL6; IL6;CXCL8;CADM1;MMP1;LUM;TNFAIP3;CXCL1
	Apoptosis	2.94E-06	1.35E-05	IL1A; IL6;GCH1;LUM;EREG;BIRC3
	IL-6/JAK/STAT3 Signaling	6.60E-05	2.53E-04	IL6; CSF2;CXCL1;CXCL3
	Interferon Gamma Response	1.40E-04	4.59E-04	IL6;GCH1;TNFAIP3;CCL2;PTGS2
	UV Response Up	6.51E-04	1.87E-03	IL6;ABCB1;GCH1;CXCL2
	Allograft Rejection	1.56E-03	3.99E-03	IL6;CCL2;ICOSLG;EREG
	Pperoxisome	2.24E-03	5.16E-03	ABCB1;CADM1;SERPINA6
Day3+IS vs Day3	TNF-alpha Signaling via NF-kB	5.49E-04	9.33E-03	IFIH1;TIPARP;SLC16A6;FOSB
	Hypoxia	5.53E-02	1.34E-01	TIPARP;STC2
	Glycolysis	5.53E-02	1.34E-01	STC2;CACNA1H
	Estrogen Response Early	5.53E-02	1.34E-01	TIPARP;STC2
	Myogenesis	5.53E-02	1.34E-01	STC2;CACNA1H
	KRAS Signaling Up	5.53E-02	1.34E-01	SLPI;SPP1
	Epithelial Mesenchymal Transition	5.53E-02	1.34E-01	SPP1;MXRA5
	Angiogenesis	6.62E-02	1.41E-01	SPP1
	Interferon Alpha Response	1.69E-01	3.00E-01	IFIH1
	PI3K/AKT/mTOR Signaling	1.81E-01	3.00E-01	MAP2K6
Day9+IS vs Day9	Epithelial Mesenchymal Transition	9.93E-06	2.98E-04	RGS4;CDH6;CXCL6;MMP1;IGFBP3;IGFBP2;MMP3;SLIT2;MXR
	Coagulation	3.79E-04	5.69E-03	THBD;SERPINB2;CFH;MMP1;C1R;MMP3
	Hypoxia	2.58E-03	2.58E-02	PPP1R3C;IGFBP3;STC2;SLC2A5;PPFIA4;CP
	TNF-alpha Signaling via NF-kB	1.23E-02	5.27E-02	CXCL6;SERPINB2;CCL4;FOSB;IFIT2
	Estrogen Response Early	1.23E-02	5.27E-02	CYP26B1;STC2;AQP3;DHRS2;PTGES
	Myogenesis	1.23E-02	5.27E-02	NQO1;PPP1R3C;IGFBP3;STC2;PPFIA4
	Complement	1.23E-02	5.27E-02	ITGAM;SERPINB2;CFH;C1R;CP
	Angiogenesis	2.52E-02	9.45E-02	CXCL6;THBD
	Interferon Alpha Response	2.91E-02	9.69E-02	IFITM1;LAMP3;IFIT2
	Estrogen Response Late	4.89E-02	1.33E-01	CYP26B1;DHRS2;ASS1;PTGES

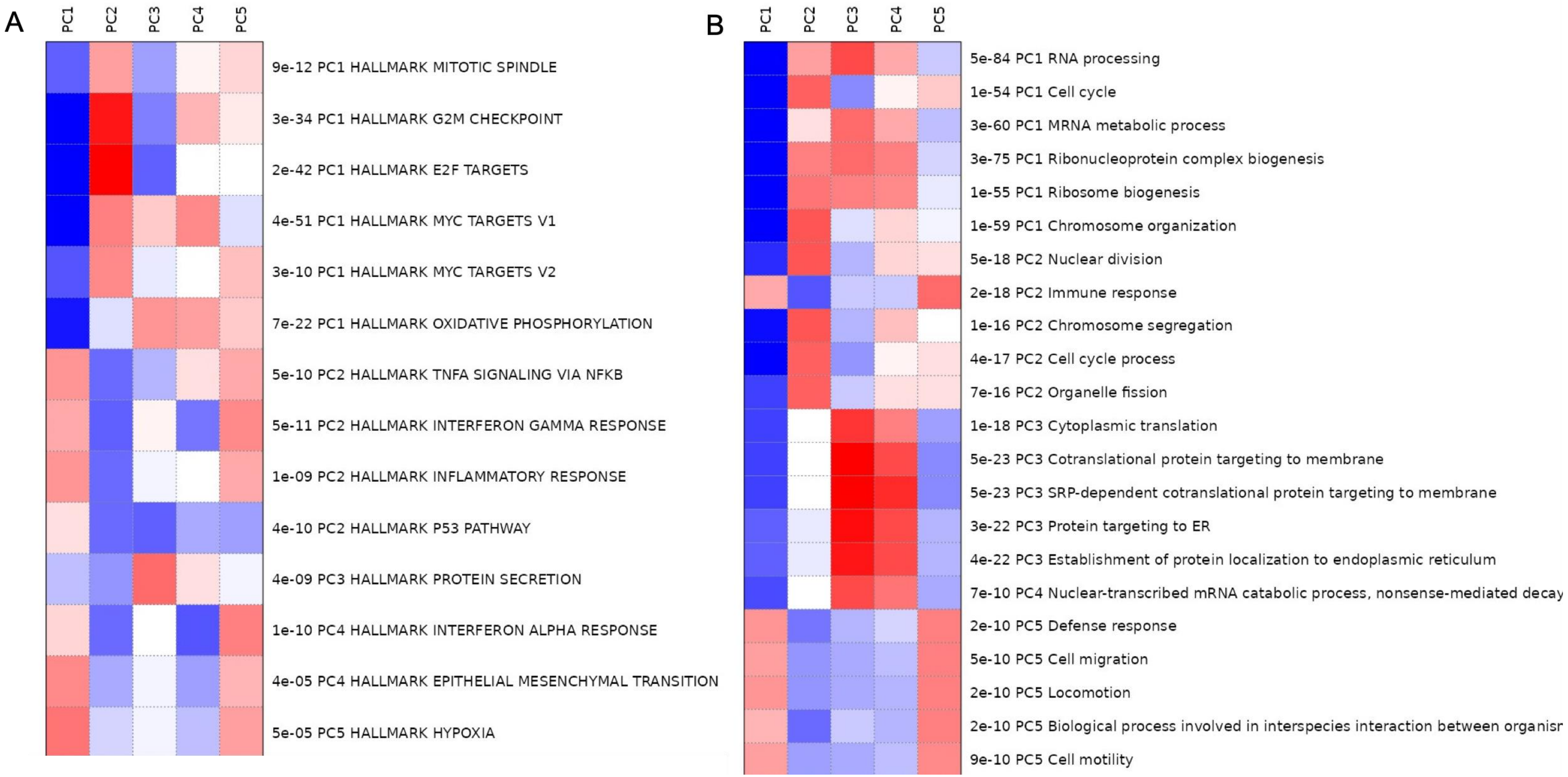

Figure S1. Pathway Analysis of PCA rotation. Pathway analysis is based on (A) MSigDB Hallmark and (B) Gene Ontology (GO) biological process. The adjusted P-values are used to rank the pathways for each of the first 5 principal components. The pathways are labeled with FDR first, followed by the principal components (PC1, PC2 and so on).). Only 6 pathways for each principal components are shown, but duplicated ones are skipped.

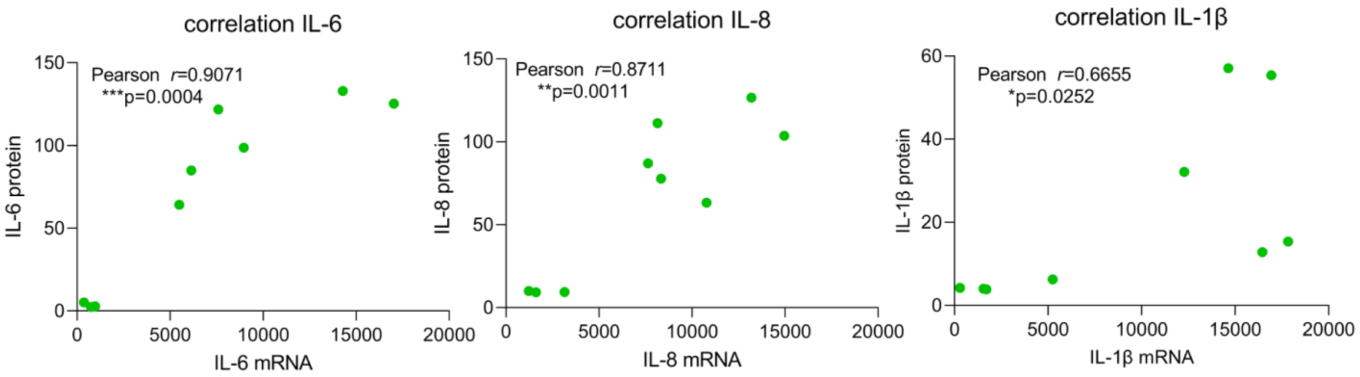

Figure S2. Correlation analysis of IL-6, IL-8,and IL-1β protein level in Figure 3 and mRNA abundance in Figure 5. Protein levels and gene counts are on day 0, day 3, and day 9 with the treatment of IS (200 µM). $P<0.05$ is considered to be statistically significant.

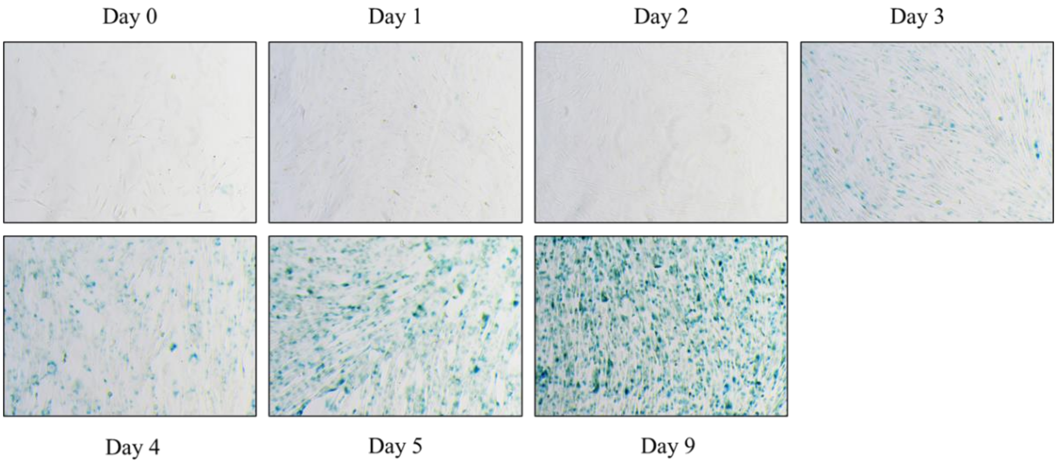

Figure S3. SA-β-gal activity in CiPTEC-OAT1 during culture at non-permissive temperature. Representative images of SA-β-gal staining in ciPTEC-OAT1 cultured for 0 through 9 days at 37 °C. Three independent experiments were performed.

Figure S4. Exposure to IS leads to sustained common senescence markers expression in ciPTEC-OAT1 at non-permissive temperature of 37℃.

Figure2 A and C														
	D0		D3				D6				D9			
laminb1 66KD	0	0	0	50	100	200	0	50	100	200	0	50	100	200
1		1,273743	1,540788	1,240701	1,487106	1,182962	1,362591	1,167913	0,898066	0,873887	0,924811	0,773353	0,825469	0,58539
2		1,332153	1,543898	1,347214	1,047683	1,10861	1,124197	1,343087	0,900824	0,754869	0,598426	0,895715	1,024614	0,501037
3		1,294441	2,366507	1,631614	1,518184	1,685858	0,959858	0,778055	0,569896	0,697054	0,77864	0,571629	0,562489	0,489033
P21 (21KD)														
1			0,717195	0,993097	1,374536	1,415576	2.458695*	2,548834	1,813102	1,596976	0,66553	1,537727	1,772006	1,160755
2			0,559451	0,885949	1,004418	0,761059	1,137537	2,281764	2,560881	1,697365	0,644766	0,950888	1,12494	0,606325
3			1,221396	1,332109	1,090661	0,819061	0,632169	0,855653	0,648725	0,806503	0,498862	0,89509	1,178016	0,709862
4			0,86138	0,863684	1,069616	1,110895	0,719731	1,169171	1,241839	1,164127	1,204798	1,202833	1,708423	1,701798

Lamin B1 66 kDa

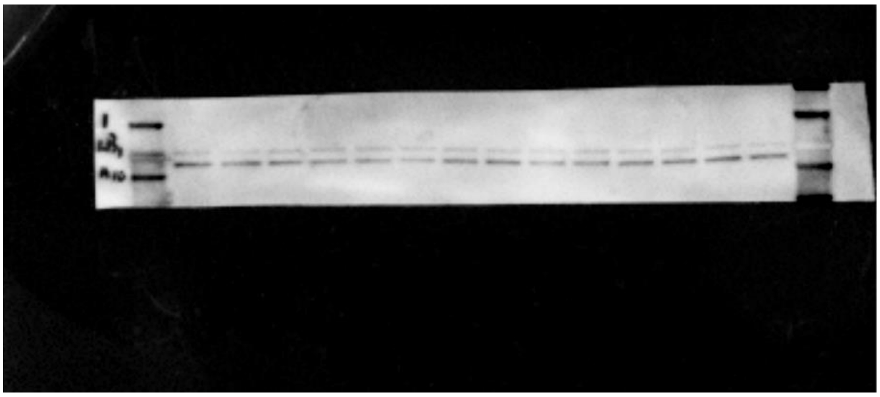

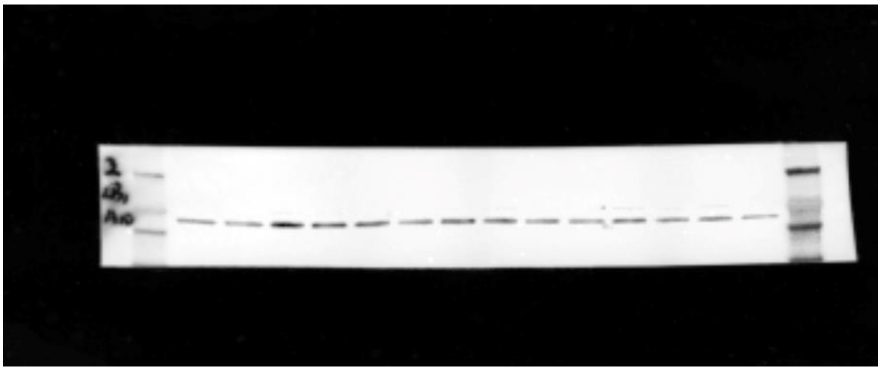

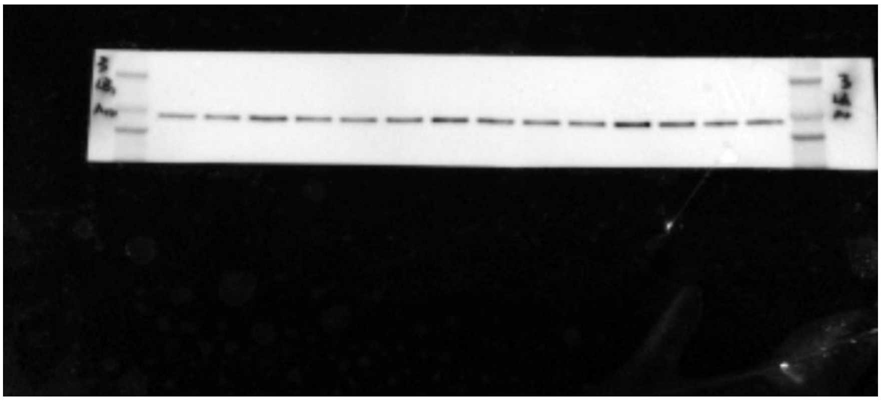

P21 (21 kDa)

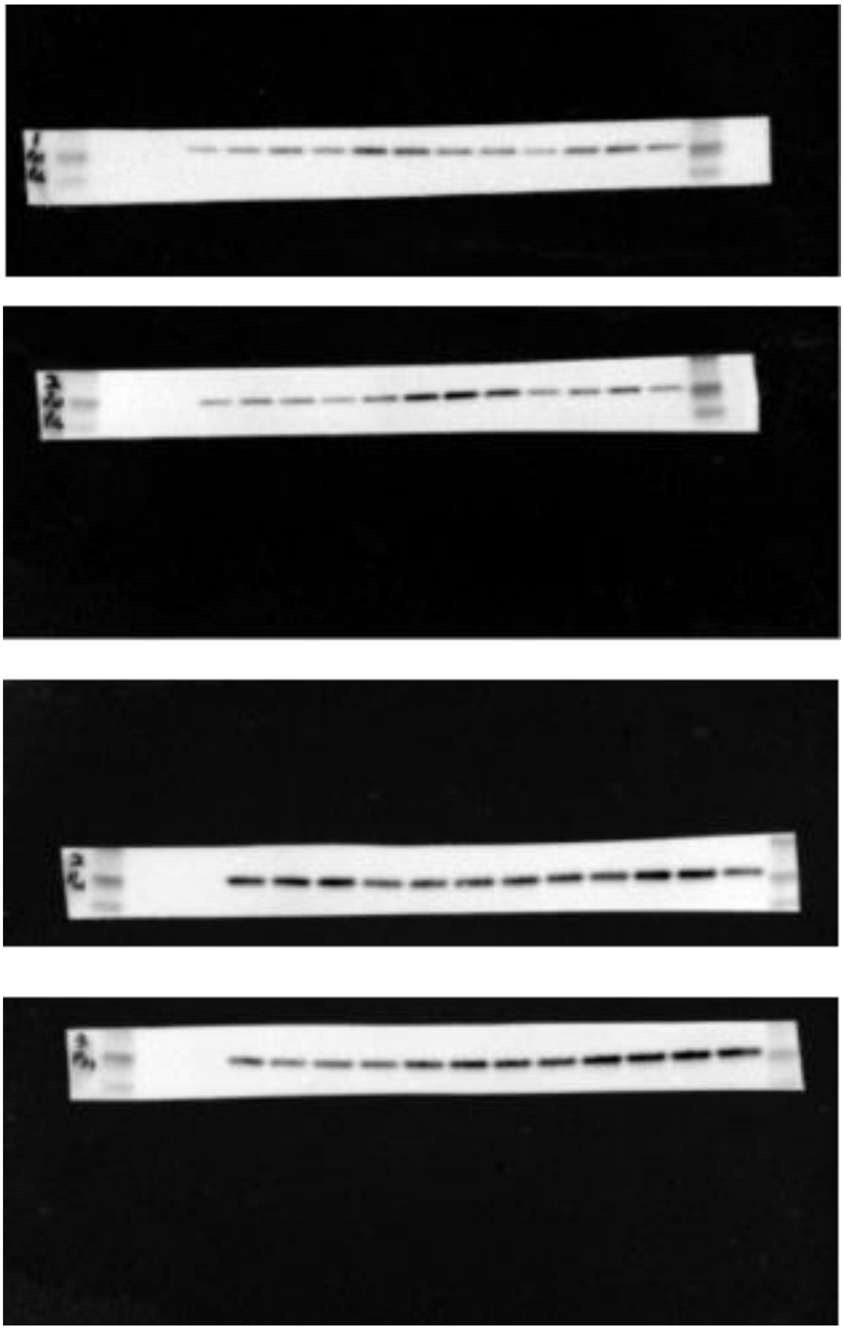

b-actin (42 kDa)

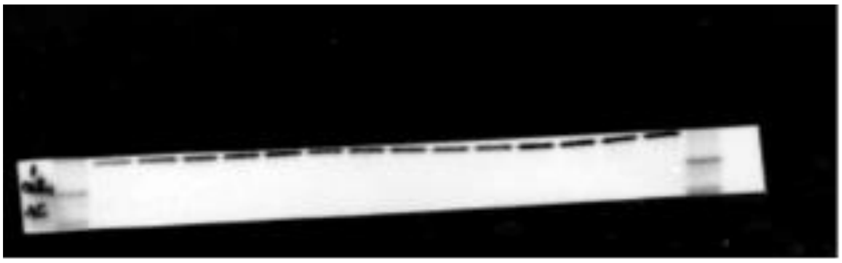

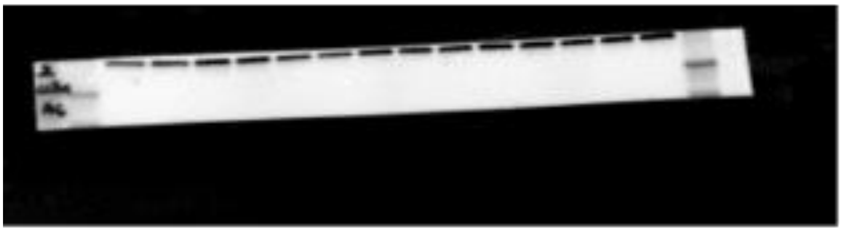

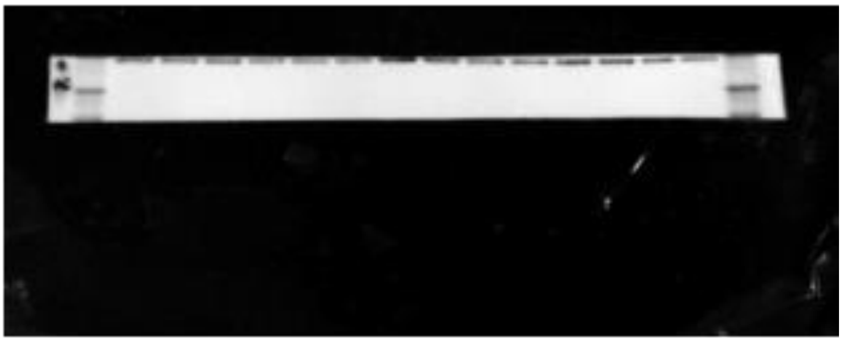

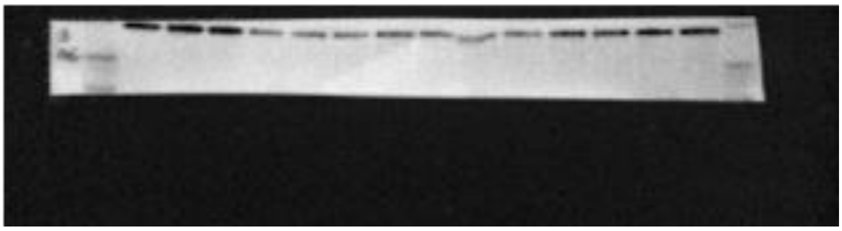

Supplement: Supplementary file 1 [file toxins-15-00242-s001.zip › toxins-2258599-supplementary.pdf]
